# Supplementary material for: Epigenetic control of adaptive or homeostatic splicing during interval-training activities
Source: Nucleic Acids Res. 2024 Apr 25;52(12):7211–24. doi: 10.1093/nar/gkae311 (PMC11229381; doi:10.1093/nar/gkae311)
Supplement: gkae311_Supplemental_File [file gkae311_supplemental_file.pdf]

Supplementary Figure Legends

S_Figure 1. Adaptive splicing upon ITD KCl treatments: pre-tested STREX, different cell lines, exome analysis and examples of RT-PCR validations. A. Pre-test for the time course using STREX splicing response to single KCl (50mM) treatment (gray) or with KCl wash-off at 6h (orange) in GH_3 cells. The STREX inclusion level before the treatment is indexed as 1. **B.** Cell-dependent adaptive splicing of STREX upon ITD (mean ± s.d., n = 3). **C**. GH_3 growth curves throughout the treatments. Note that the STREX showed homeostatic splicing response to depolarization in GH_3 and LA-N-5 cells here but adaptive splicing in the PC12 and N1E cells, with basal levels of 50%, 45%, 11% and 30% in the NT samples of the respective cell lines. NT: untreated. The changes in STREX inclusion levels induced by both the 1st and 6th KCl treatments were found to be statistically significant across all examined cell lines ($p < 0.05$). The only exception was the lack of significant change in STREX inclusion level in N1E cells following the 6th KCl treatment. **D**. Scatter plot of the exon inclusion level (normalized average reads, n = 3) between the 1st and 6th KCl-treated samples by DEXSeq analysis. RPKM, reads per kilobase per million. **E**. DAVID functional clusters of 1,204 genes whose exons changed between the 6th and 1st KCl-treated samples based on DEXSeq and MATS analysis. **F**. Schematic diagram of ideal changes according to the three primary splicing response patterns observed, with KCl-downregulated exons as examples. **G**. Representative agarose gels of RT-PCR-validated exons of the three primary splicing response patterns. Boxes in grey: alternative; white: constitutive exons; arrowhead: primers; In: inclusion of alternative exon; Δ: net change of percent exon inclusion by KCl treatment; -: PCR negative control; *Gapdh*: RNA loading control. **H**. Bar graphs of the net percent changes of the exon inclusion upon single (1st) or ITD (6th) KCl treatment (mean ± s.d., n

⩾ 3). Open circles: before, and filled circles: after KCl treatment. ns, not significant; **: $p < 0.01$; ****: $p < 0.0001$.

S_Figure 2. Effect of signaling pathway inhibitors on the adaptive splicing of a reporter exon. Scatter plot of tyrphostin AG 1288, nifedipine and SB202109 treatment on the depolarization effect of the 6th KCl treatment on the reporter exon in N1E cells. In this assay, SB-202190 (p38 MAPK pathway inhibitor), Tyrphostin AG 1288 (Tyrosine kinases pathway inhibitor) and nifedipine (Ca2+/CaMKIV pathway inhibitor) were tested. One day after transfection of the reporter plasmid DUP175-CaRRE-D56 (CaRRE: a CaMK IV-responsive RNA element, 53-nucleotide intron + 1-nucleotide STREX exon), cells were treated with 50mM KCl and respective inhibitors (10µM each) for 6h, then washed and added back with fresh complete growth medium till the next treatment 18h later as in Fig. 1A. The assays were carried out in duplicates. Arrows: primers. The three inhibitors differentially affected both the 1st and 6th KCl effects on the reporter exon. Particularly, the nifedipine effect is consistent with a role of L-type calcium channels and CaMKIV as in our previous report (Xie, et al., *RNA*, 2005 Dec 11(12):1825-1834).

S_Figure 3. Exonic DNA methylation (EDM) levels versus splicing changes and other examples of the adaptive or homeostatic splicing of synaptic exons disrupted by 5-azaC. **A-B.** Scatter plots of the genome-wide EDM levels (A) or their index of net changes (B, per kilobases of exon DNA) and the transcriptome-wide fold changes (FC, ratio of +/- 5-azaC) of exon usage induced by 5-azaC in the 6th KCl-treated GH3 cells. n = 22,361 (mCpG) and 28,077 (mCpH) exons. The total methylation level of exon DNA was calculated by the average methylation ratio (0-1) of

CpG or CpH multiplied by its total number of CpG or CpH sites. In (A), examples of studied exons are highlighted: Mapt exon 6 with a yellow circle, Epb41l3 exon 15 with a blue circle, Mapt exon 10 with a green circle, and Mapt exon 7a with a black circle. **C**. Bar graphs of net percent changes of exon inclusion upon single (1st) or ITD (6th) KCl treatment (mean ± s.d., $n \geqslant 3$) with or without 5-azaC. In brackets are the corresponding human exon rank/numbers. Homeo: homeostatic; DS: desensitized; HS: hypersensitive; R: reversed. ns: not significant, *: $p < 0.05$, **: $p < 0.01$, ***: $p < 0.001$. The exon numbers are based on reference transcripts in the UCSC Genome Rat Jul. 2014 (RGSC 6.0/rn6) Assembly: Epb41l3 exon 15, NM_053927.1; Kidins220 exon 26, NM_053795.1; Mapt exon 6, M84156, equivalent to human MAPT exon 4a (NM_001123066.3, GRCh38/hg38); Mapt exon 10, M84156, equivalent to human MAPT exon 10 (NM_001123066.3, GRCh38/hg38); Nrg1 exon 12, NM_001271128.1; Dlg1 exon 20a, NM_012788.1, between exons 20 and 21; Phldb1 exon 10, X74226; Mapt exon 7a, M84156, between exons 7 and 8, equivalent to human MAPT exon 6 (NM_001123066.3, GRCh38/hg38). **D**. Scatter plot of the exon mC (mCpG or mCpH) levels and corresponding ratios of exon usage (log2) by RT-PCR results, with/without 5-azaC in the 6th KCl-treated GH3 samples (n = 31 exons in total).

S_Figure 4. An example of the aberrant splicing of constitutive exons upon disruption of EDM by 5-azaC and its aggravation by repeated depolarization in GH_3 cells. A. Diagram of the *Dlg1* splicing patterns observed after 5-zazC and ITD of GH_3 cells. *Dlg1* exon numbering is based on the reference sequence NM_012788.1 of the RGSC 6.0/rn6 Assembly. *Dlg1* exon 20a is an alternative exon, while exons 19, 20, 21, 22, and 24 are constitutive exons in untreated GH_3 cells. **B.** Agarose gels of RT-PCR products upon repeated KCl treatments with/without 5-azaC (50µM). Asterisk: Heteroduplex of *Dlg1-1* and *Dlg1-2*. Products were confirmed by Sanger

sequencing. Circle or hollow triangle: before KCl treatment; dots or solid triangle: after KCl treatment. **C.** Dose-dependent effects on the aberrant splicing of *Dlg1* constitutive exons 21 (*Dlg1*-3 variant) and 20 (*Dlg1*-4 variant) induced by increasing concentrations of 5-azaC (0, 1nM, 1µM, 10µM, 20µM or 50µM). **D.** EDM changes of the *Dlg1* constitutive exons in the 6th KCl-treated cells with (black) or without (red) 5-azaC (50µM) treatment. Upper: mCpG; Lower: mCpH.

S_Figure 5. More examples of 5-azaC induced adaptively aberrant splicing of synaptic genes upon ITD. Agarose gels of RT-PCR of RNA from cells upon repeated KCl treatments with or without pre-treatment by 5-azaC (50µM) in GH_3 cells. Open boxes: constitutive exons; Filled boxes: alternative exons; Black arrowheads: primers; Red arrowheads: PCR products with aberrant exon skipping or intron usage. Assay in triplicates. *: Product of unknown identity. The exon ranking numbers are by the reference transcripts: *Nsmf* exon 9, NM_057190.2; *Gphn* exon 7a, NM_022865.3, between exons 7 and 8; *Gipr* intron 10, NM_012714.1.

S_Figure 6. Aberrant splicing of *Prolactin* induced by 5-azaC upon ITD in GH_3 pituitary cells, accompanied by mRNA transcript level change and EDM disruption. A. RT-PCR of RNA from cells upon ITD with or without 5-azaC. Without 5-azaC treatment, there are no significant change of splicing and the mRNA level of Gh1 and Prl upon either the 1st or the 6th KCl, suggesting that the cells have kept their endocrine identities. With 5-azaC treatment, the the *Prolactin* gene exons 2-3 were aberrantly skipped, accompanied by reduced *Prl* transcript levels and EDM changes upon ITD. *Gapdh* (Glyceraldehyde-3-Phosphate Dehydrogenase), RNA loading control. **B.** Bar graph of the normalized level of the *Prl* transcript. ns: not significant, *: *p*

< 0.05; ***: $p < 0.001$. **C.** Diagram of the *Prl* variants as well as the average EDM level of each exon upon ITD with or without 5-azaC. Consistently, the EDM of these skipped exons was disrupted by 5-azaC with hypermethylation in exons 2 (from 50% to 87.5%) and 3 (from 53% to 72%), in comparison to the hypomethylation in exons 4 and 5 (from 77% to 45%).

Note: the *Prl* promoter is hypermethylated (mCG and mCH, mainly mCH) from 42% to 83% upon 5-azaC treatment, suggesting transcriptional inhibition by 5-azaC via hypermethylation of its promoter. In comparison, consistent with the unchanged transcript level and stable splicing, the average methylation level of the *Gh1* promoter and EDMs weren't significantly changed by 5-azaC.

S_Figure 7. MeCP2 mutation effect on the aberrant splicing of other hippocampal genes *in vivo*. A. Scatter plots of the EDM mC levels of wild type mice and fold changes of corresponding exon usage with/without kainic acid treatment in the hippocampus of wild type (blue) or *Mecp2*-null (brown) mice, by analysis of the raw reads from the datasets by Osenberg or Guo respectively. The FCs less than one are displayed as -1/FC to illustrate the shift from 1 (or -1) in the *Mecp2*-null mice. Note the further shift of brown (mutant) dots overall away from the midline compared to the blues WT ones of the same group of exons. n = 1,719 exons for both mCpG and mCpH. **B-D.** Agarose gels (**B**) of RT-PCR products of splice variants, their mean (± s.d.) exon inclusion levels (**C**), and a bar graph (**D**) of the net changes of exon usage upon each *MECP2* mutation (patient) from the mean of controls. The MeCP2 IVS3-2A>G mutation consistently exhibited the smallest splicing changes compared to other mutations including R255X, A201V and T158M in different adaptive exons in the hippocampus samples of *MECP2*-mutated Rett syndrome patients, suggesting mutation-dependent differential effects on the extent of aberrant splicing. NA: mutation

information not available. Arrowhead: IVS3-2A->G samples. The numbering of human exons is based on the following transcripts: *EPB41L3* exon 15, NM_012307.4, equivalent to the *Epb41l3* exon 15 (NM_053927.1); *KIDINS220* exon 26, NM_001348729.2, equivalent to the *Kidins220* exon 26 (NM_053795.1); *MAPT* exon 10, NM_001123066.3, equivalent to *Mapt* exon 10 (M84156); *GPHN* exon 8, NM_020806.4, equivalent to *Gphn* exon 6a (NM_022865.3). MAPT exon 6 (4a) and DLG1 exon 19 were also tested but without a significant difference observed.

S_Figure 8. Differentially expressed genes upon ITD in GH_3 pituitary cells. A. Venn diagram showing the number of differentially expressed genes upon a single (1st) or repeated (6th) KCl treatments. By filtering with average normalized counts > 50, 4,607 of 32,662 detected genes were selected for further analysis. Among them, 2,654 genes were identified with differential expression (>1.1FC) upon ITD compared to the single treatment samples, including ones (~24.6%, 655/2654) that barely even responded to the 1st treatment. **B.** Heatmap showing that 1,024 genes exhibited differential changes (>1.1FC, FDR<0.5, aveNormalized counts > 50) in transcript level between the 6th and 1st KCl treated samples by edgeR analysis. FC, fold change; red, upregulation; blue, downregulation. **C.** Volcano plot displaying the up-(red) and down-regulated (blue) genes (n = 238 genes, > 2FC, 1st vs 6th KCl treatment) upon ITD (FDR > 0.5, aveNormalized counts > 50). Black arrows indicate the position of hnRNP L and LL below the threshold, which also had no significant changes by RT-PCR in the MeCP2 knockdown cells. **D.** DAVID functional clustering analysis of the 238 differentially expressed genes, ranked by -Log10 of the *p* values. **E.** Bar graph of representative RT-PCR-validated splicing factor or DNA methylation genes with adaptive changes upon repeated KCl treatments.

S_Fig. 1

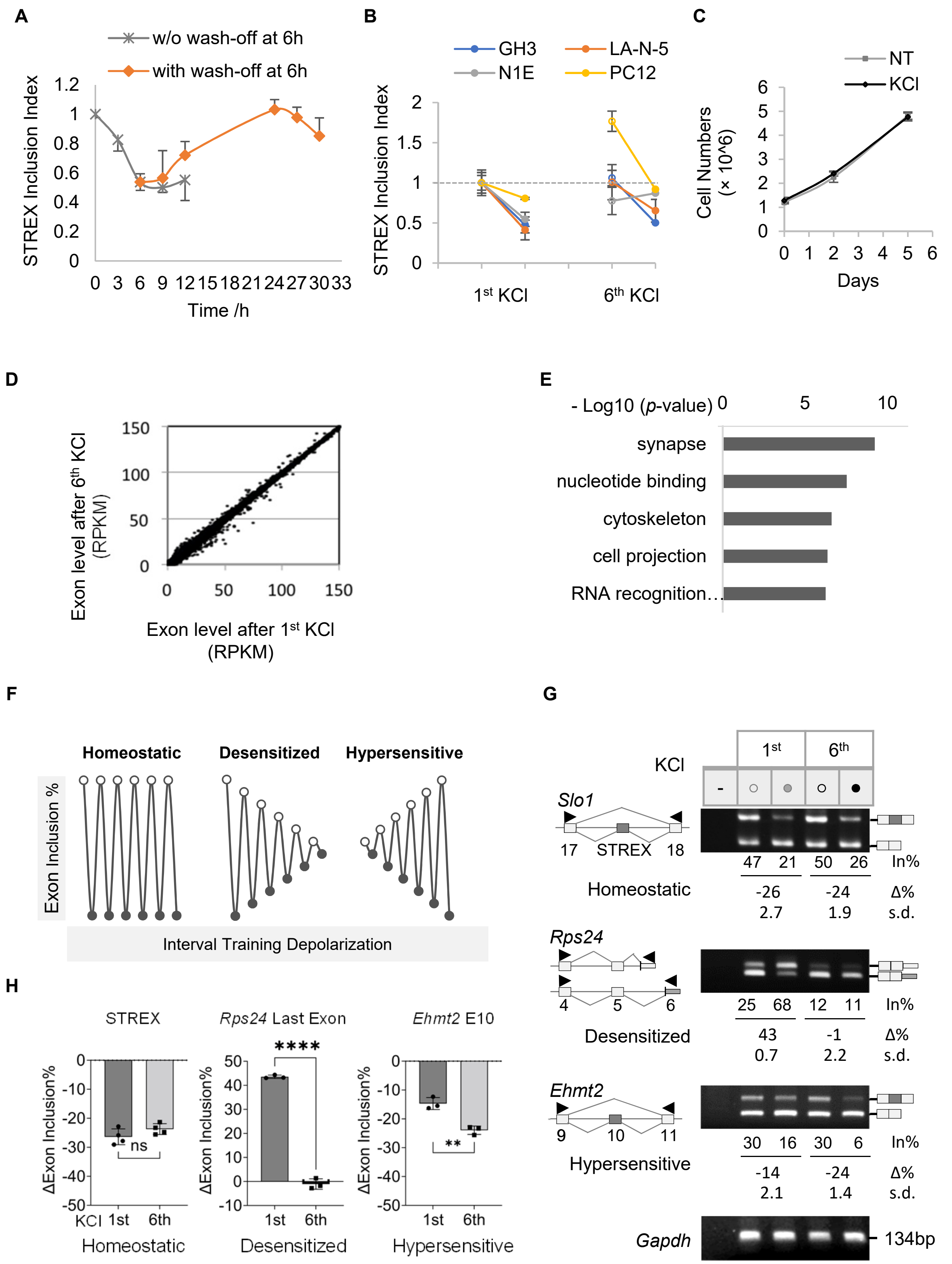
A
w/o wash-off at 6h
with wash-off at 6h
STREX Inclusion Index
Time /h
B
GH3
LA-N-5
N1E
PC12
STREX Inclusion Index
1st KCl
6th KCl
C
NT
KCl
Cell Numbers (× 10^6)
Days
D
Exon level after 6th KCl (RPKM)
Exon level after 1st KCl (RPKM)
E
- Log10 (p-value)
synapse
nucleotide binding
cytoskeleton
cell projection
RNA recognition…
F
Homeostatic
Desensitized
Hypersensitive
Exon Inclusion %
Interval Training Depolarization
G
KCl
1st
6th
Slo1
17 STREX 18
47 21 50 26 In%
Homeostatic
-26 -24 Δ%
2.7 1.9 s.d.
Rps24
4 5 6
25 68 12 11 In%
Desensitized
43 -1 Δ%
0.7 2.2 s.d.
Ehmt2
9 10 11
30 16 30 6 In%
Hypersensitive
-14 -24 Δ%
2.1 1.4 s.d.
Gapdh
134bp
H
STREX
Rps24 Last Exon
Ehmt2 E10
ΔExon Inclusion%
ns
****
**
KCl 1st 6th
Homeostatic
Desensitized
Hypersensitive

S_Fig.2

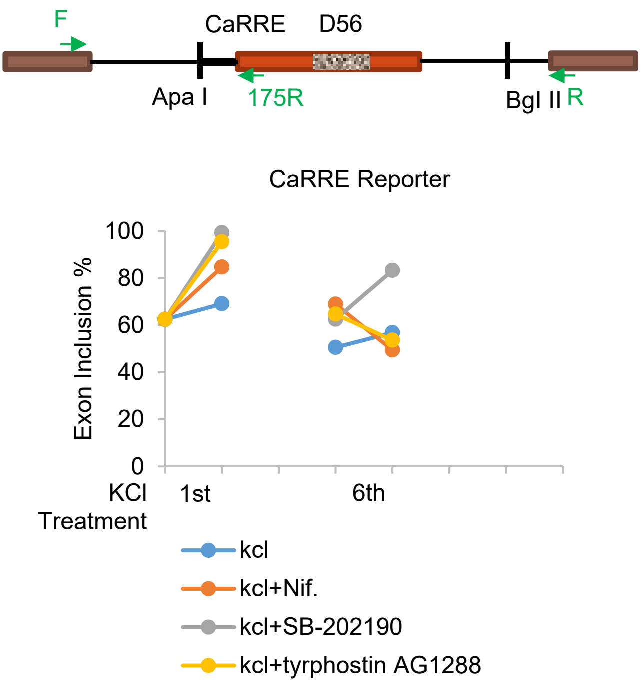
F
CaRRE
D56
Apa I
175R
Bgl II
R
CaRRE Reporter
Exon Inclusion %
100
80
60
40
20
0
KCl
Treatment
1st
6th
kcl
kcl+Nif.
kcl+SB-202190
kcl+tyrphostin AG1288

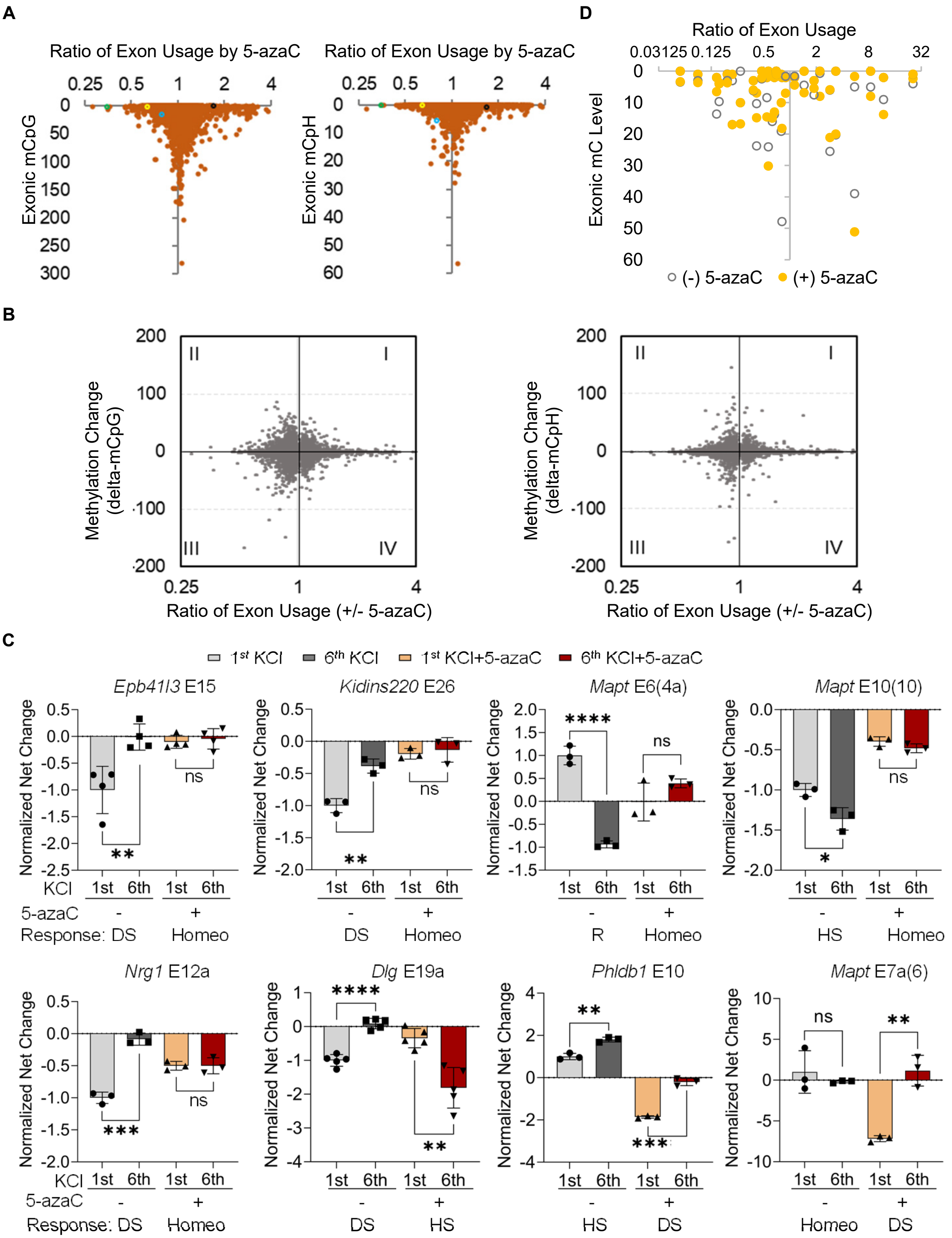
A
Ratio of Exon Usage by 5-azaC
Exonic mCpG
Ratio of Exon Usage by 5-azaC
Exonic mCpH
D
Ratio of Exon Usage
Exonic mC Level
(-) 5-azaC
(+) 5-azaC
B
Methylation Change (delta-mCpG)
Ratio of Exon Usage (+/- 5-azaC)
Methylation Change (delta-mCpH)
Ratio of Exon Usage (+/- 5-azaC)
II
I
III
IV
C
1st KCl
6th KCl
1st KCl+5-azaC
6th KCl+5-azaC
Epb41l3 E15
Kidins220 E26
Mapt E6(4a)
Mapt E10(10)
Nrg1 E12a
Dlg E19a
Phldb1 E10
Mapt E7a(6)
Normalized Net Change
KCl
5-azaC
Response:
DS
Homeo
R
HS
ns

S_Fig.4

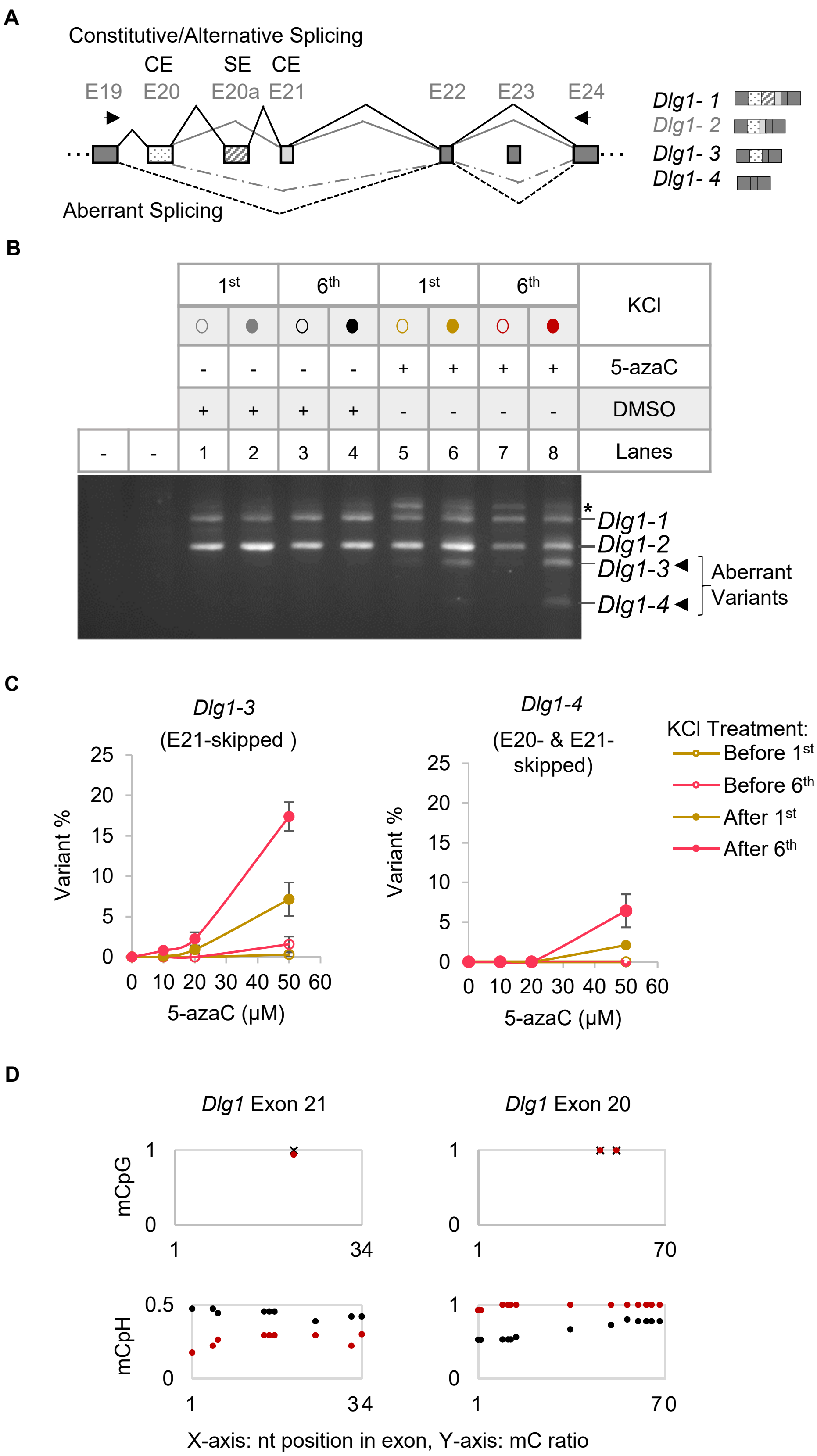
A
Constitutive/Alternative Splicing
CE
SE
CE
E19
E20
E20a
E21
E22
E23
E24
Aberrant Splicing
Dlg1- 1
Dlg1- 2
Dlg1- 3
Dlg1- 4
B
1st
6th
1st
6th
KCl
5-azaC
DMSO
Lanes
1
2
3
4
5
6
7
8
*
Dlg1-1
Dlg1-2
Dlg1-3
Dlg1-4
Aberrant Variants
C
Dlg1-3
(E21-skipped)
Dlg1-4
(E20- & E21- skipped)
Variant %
5-azaC (μM)
KCl Treatment:
Before 1st
Before 6th
After 1st
After 6th
D
Dlg1 Exon 21
Dlg1 Exon 20
mCpG
mCpH
X-axis: nt position in exon, Y-axis: mC ratio

S_Fig.5

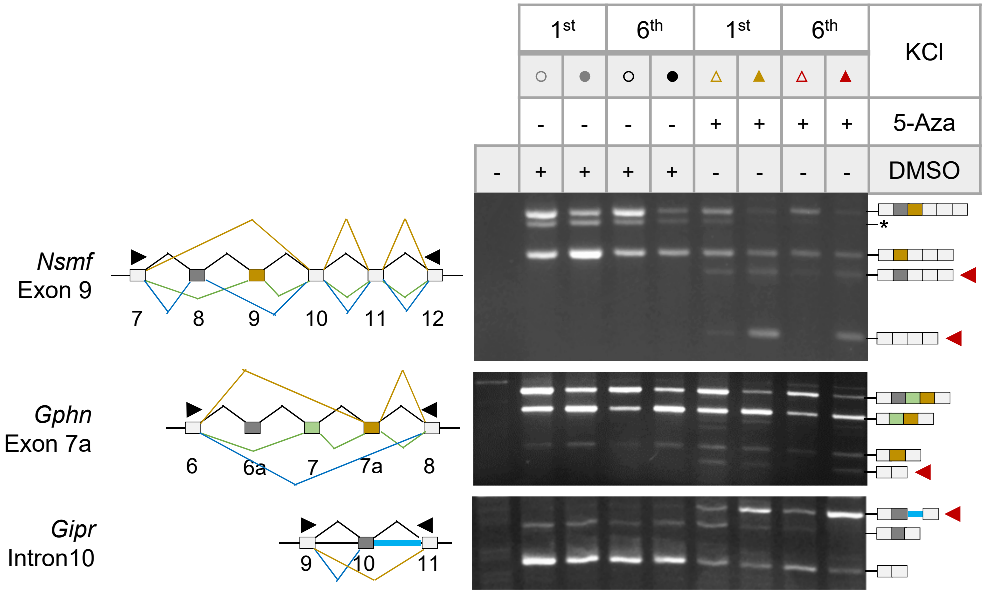
1st
6th
1st
6th
KCl
5-Aza
DMSO
Nsmf
Exon 9
7
8
9
10
11
12
Gphn
Exon 7a
6
6a
7
7a
8
Gipr
Intron10
9
10
11
*

S_Fig.6

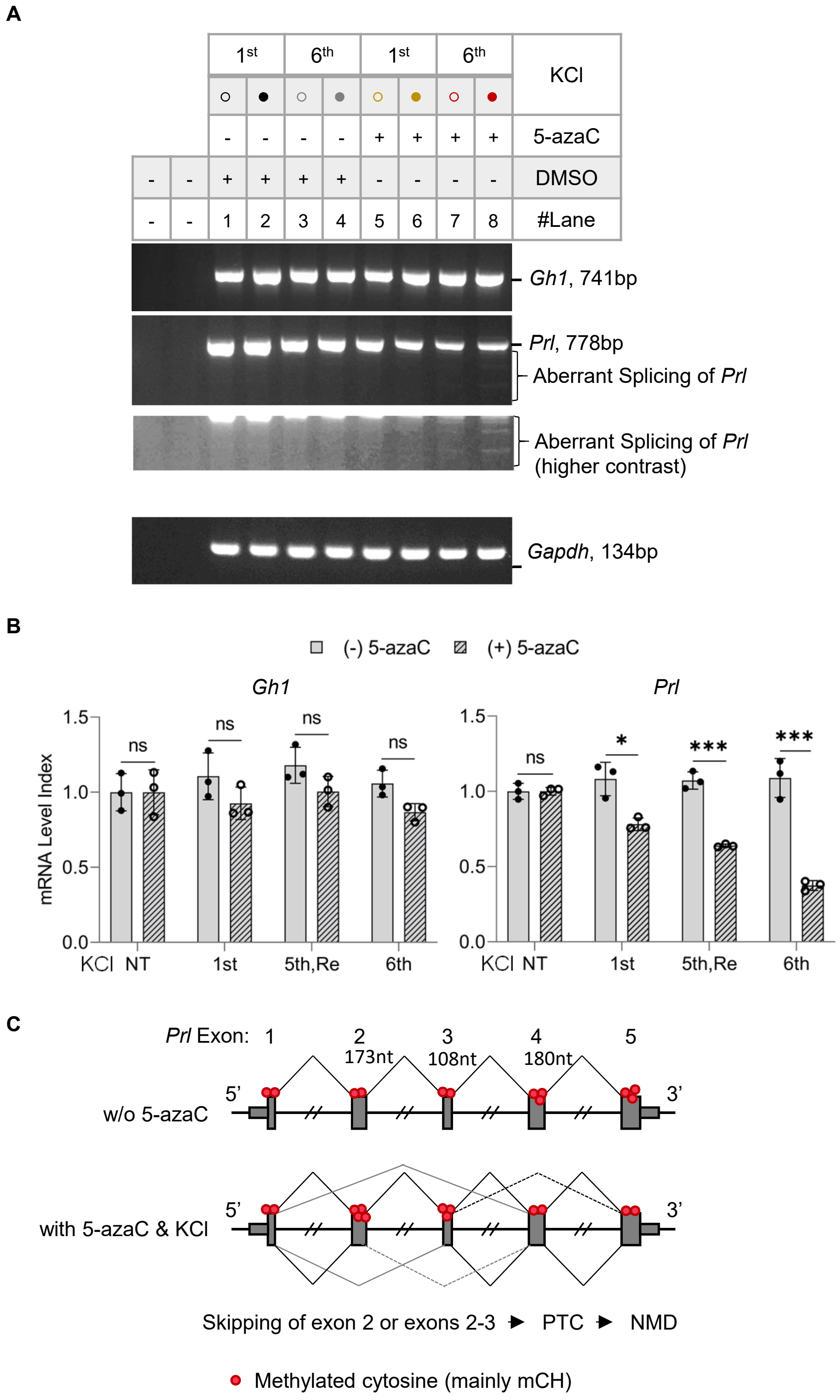
A
1st
6th
1st
6th
KCl
5-azaC
DMSO
#Lane
- - + + + + - - - -
- - 1 2 3 4 5 6 7 8
Gh1, 741bp
Prl, 778bp
Aberrant Splicing of Prl
Aberrant Splicing of Prl (higher contrast)
Gapdh, 134bp
B
(-) 5-azaC
(+) 5-azaC
Gh1
Prl
mRNA Level Index
1.5
1.0
0.5
0.0
ns
*
***
KCl NT
1st
5th,Re
6th
C
Prl Exon: 1 2 3 4 5
173nt
108nt
180nt
5'
3'
w/o 5-azaC
with 5-azaC & KCl
Skipping of exon 2 or exons 2-3 ► PTC ► NMD
Methylated cytosine (mainly mCH)

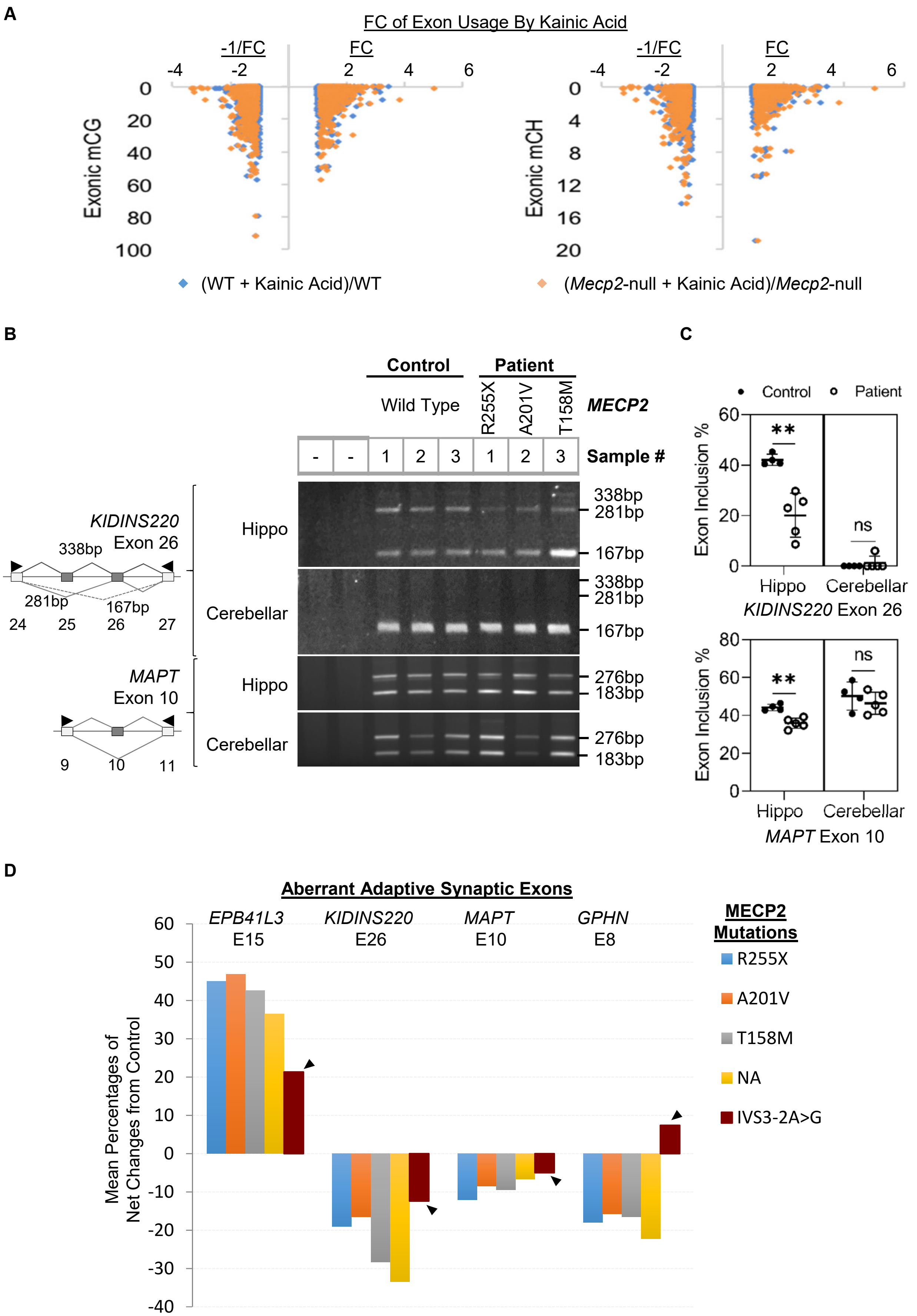
A
FC of Exon Usage By Kainic Acid
-1/FC
FC
Exonic mCG
Exonic mCH
(WT + Kainic Acid)/WT
(Mecp2-null + Kainic Acid)/Mecp2-null
B
Control
Patient
Wild Type
R255X
A201V
T158M
MECP2
Sample #
KIDINS220
Exon 26
338bp
281bp
167bp
24
25
26
27
MAPT
Exon 10
9
10
11
Hippo
Cerebellar
276bp
183bp
C
Control
Patient
Exon Inclusion %
Hippo
Cerebellar
KIDINS220 Exon 26
MAPT Exon 10
**
ns
D
Aberrant Adaptive Synaptic Exons
EPB41L3 E15
KIDINS220 E26
MAPT E10
GPHN E8
Mean Percentages of Net Changes from Control
MECP2 Mutations
R255X
A201V
T158M
NA
IVS3-2A>G

S_Fig. 8

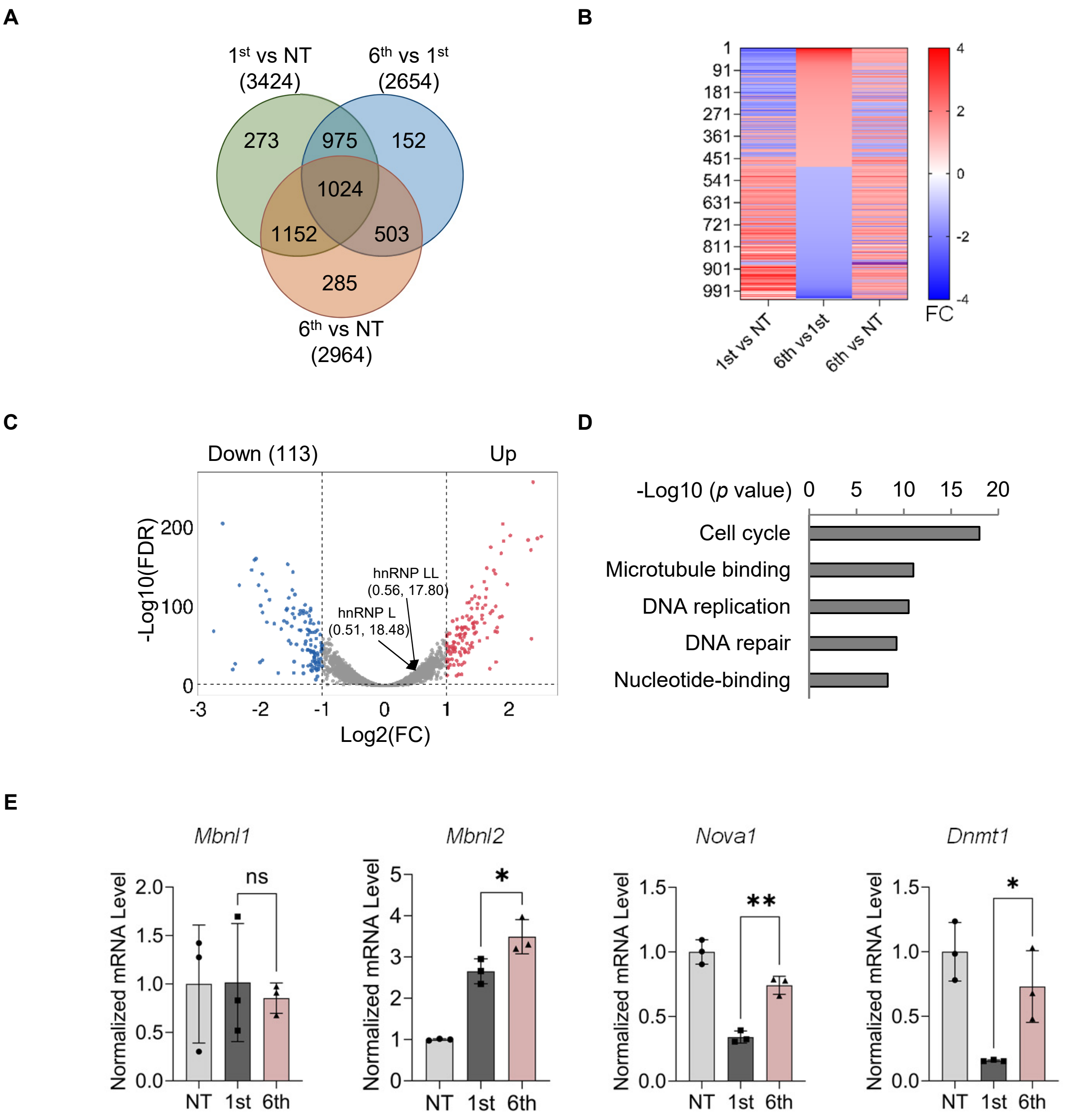
A
1st vs NT (3424)
6th vs 1st (2654)
6th vs NT (2964)
273
975
152
1024
1152
503
285
B
1st vs NT
6th vs1st
6th vs NT
FC
C
Down (113)
Up
hnRNP LL (0.56, 17.80)
hnRNP L (0.51, 18.48)
-Log10(FDR)
Log2(FC)
D
-Log10 (p value)
Cell cycle
Microtubule binding
DNA replication
DNA repair
Nucleotide-binding
E
Mbnl1
Mbnl2
Nova1
Dnmt1
Normalized mRNA Level
ns
NT 1st 6th

S_Table I. **Exons of synaptic genes regulated by both hnRNP L and 5-azaC, analyzed with DEXSeq and their methylation changes determined by BSMAP.** In the brackets are the commonly referenced exon numbers of human *MAPT*. (Hypo: mC level reduced, Hyper: mC level increased after 5-azaC treatment, and 0 indicates no change overserved. FC: fold change.)

Gene Name	Exon#	Gene Bank	FC by 5-azaC	FC by shL	Exonic mCpH level	Exonic mCpG level
Mapt	E7a (6)	M84156	1.79	-1.32	hyper	hypo
Syne1	E90	MK681777	1.56	-1.20	hyper	0
Gabbr1	E19	NM_031028.3	1.27	1.32	hypo	0
Dlg1	E20a	NM_012788.1	-1.21	1.75	hyper	0
Cacna1d	E46	NM_017298.1	-1.22	-1.64	hyper	0
Epb41l3	E15	NM_053927.1	-1.27	-3.70	hyper	0
Mapt	E6 (4a)	M84156	-1.33	1.48	hyper	hyper
Cacna1d	E34	NM_017298.1	-1.41	-1.92	hyper	0
Gphn	E6a	NM_022865.3	-1.47	1.23	hypo	0
Clasp2	E17	NM_053722.2	-1.52	-1.18	hypo	0
Kifc2	E14	NM_198752.3	-1.54	-1.82	hypo	hypo
Nrg1	E12	NM_001271128.1	-1.59	1.20	hyper	0
Snap91	E29	NM_031728.1	-1.72	-3.03	NA	NA
Phldb1	E10	X74226	-1.89	-3.03	0	hypo
Mapt	E10	M84156	-2.13	1.21	hypo	0

S_Table II. Changes in gene expression of splicing factors following repeated KCl treatment (>1.5-Fold Change between the 1st and 6th KCl treatment) as Identified by RNAseq.

ENSEMBL ID	Gene Name	FC (1st KCl vs NT)	FC (6th vs 1st KCl)	FC (6th KCl vs NT)
ENSRNOG00000008450	LOC100359539	-5.63	5.11	-1.10
ENSRNOG00000045752	Rrm1	-3.15	3.71	1.17
ENSRNOG00000031440	Nova1	-1.66	2.22	1.33
ENSRNOG00000014720	Srbd1	-1.86	1.82	-1.02
ENSRNOG00000002292	Hnrnpd	-3.23	1.73	-1.86
ENSRNOG00000016507	LOC687679	-1.51	1.72	1.138
ENSRNOG00000036839	Hnrnpa1	-1.97	1.69	-1.16
ENSRNOG00000008746	Dtd1	-3.02	1.67	-1.81
ENSRNOG00000004967	Snrpb2	-1.88	1.66	-1.13
ENSRNOG00000015844	Snrpd2	-1.37	1.60	1.16
ENSRNOG00000050323	Srsf1	-2.01	1.59	-1.26
ENSRNOG00000031127	Snrpe	-1.43	1.58	1.11
ENSRNOG00000011448	Eri1	-1.46	1.56	1.07
ENSRNOG00000045568	Rbm14	-2.05	1.55	-1.32
ENSRNOG00000010737	Mbnl2	1.81	1.55	2.81
ENSRNOG00000023360	Fus	-2.95	1.54	-1.91
ENSRNOG00000020683	Hnrnpul1	-1.85	1.54	-1.20
ENSRNOG00000001501	Snrpa	-1.31	1.54	1.17
ENSRNOG00000005556	Snrpf	-1.76	1.53	-1.15
ENSRNOG00000015914	U2af2	-1.65	1.52	-1.08
ENSRNOG00000002352	Tsen15	-1.15	1.5	1.33
ENSRNOG00000011910	Hnrnpr	-1.75	1.52	-1.16
ENSRNOG00000013545	Polr2e	-1.21	1.50	1.24
ENSRNOG00000004521	Prpf39	2.45	-1.50	1.63
ENSRNOG00000046000	Clasrp	1.91	-1.51	1.26
ENSRNOG00000002914	Trmt1	1.71	-1.58	1.08
ENSRNOG00000007629	Sf3a3	1.21	-1.61	-1.33
ENSRNOG00000032441	Pan2	2.61	-1.64	1.58
ENSRNOG00000048495	Rrp12	1.58	-1.73	-1.09
ENSRNOG00000036802	Snhg11	4.64	-2.10	2.21
ENSRNOG00000015411	Apobec1	23.08	-3.75	6.16

S_Table III. Changes in gene expression of splicing factors following 5-azaC treatment (>2-Fold Change) as Identified by RNAseq.

ENSEMBL ID	Gene Name	Fold Change	P value
ENSRNOG00000006888	Gcfc2	4.37	1.13E-262
ENSRNOG00000048193	Hnrnph3	4.07	5.6804E-101
ENSRNOG00000046000	Clasrp	3.90	1.47E-193
ENSRNOG00000011932	Snrpa1	3.52	7.41E-163
ENSRNOG00000009156	Tra2a	3.36	1.31E-138
ENSRNOG00000002036	Paxbp1	3.35	6.84E-91
ENSRNOG00000025629	Prpf3	3.25	2.55E-182
ENSRNOG00000053070	LOC108350566	3.13	5.55E-125
ENSRNOG00000010827	Ptbp2	2.94	1.71E-86
ENSRNOG00000001996	Ythdc1	2.92	3.35E-167
ENSRNOG00000015332	Thoc1	2.78	1.74E-116
ENSRNOG00000016087	Hars2	2.72	9.25E-150
ENSRNOG00000003399	Hnrnph1	2.66	1.55E-139
ENSRNOG00000006655	Plrg1	2.49	2.99E-68
ENSRNOG00000017086	Zfp341	2.46	1.23E-36
ENSRNOG00000004061	Pnn	2.42	8.60E-86
ENSRNOG00000050741	Rbm4	2.29	1.35E-62
ENSRNOG00000017310	Rnpc3	2.25	1.41E-38
ENSRNOG00000027761	Htatsf1	2.23	1.62E-85
ENSRNOG00000001783	Tra2b	2.16	2.11E-80
ENSRNOG00000001397	Rbm19	2.14	2.60E-54
ENSRNOG00000009451	Prpf38a	2.11	5.78E-72
ENSRNOG00000014833	Smndc1	2.10	8.48E-69
ENSRNOG00000024372	Cwf19l2	2.08	9.71E-71
ENSRNOG00000005218	Sf3a1	2.00	1.54E-81
ENSRNOG00000014908	Sf3b5	-2.04	1.45E-62
ENSRNOG00000030374	Lsm6l1	-2.11	3.89E-10
ENSRNOG00000016507	Snrpg	-2.17	5.52E-73
ENSRNOG00000005556	Snrpf	-2.25	6.27E-64
ENSRNOG00000047628	Khsrp	-2.25	3.25E-105
ENSRNOG00000048725	Lsm2	-2.29	5.05E-65
ENSRNOG00000001163	Srsf9	-2.45	2.62E-101
ENSRNOG00000019552	Lsm7	-2.54	2.61E-50
ENSRNOG00000008639	Pabpc1	-2.63	7.01E-168
ENSRNOG00000015844	Snrpd2	-2.67	3.79E-97
ENSRNOG00000036839	Hnrnpa1	-2.78	3.29E-155
ENSRNOG00000023177	Esrp2	-2.82	1.12E-101
ENSRNOG00000001060	Snrnp35	-2.93	1.71E-84
ENSRNOG00000032232	Snrpg	-3.34	1.31E-41
